# IL-17A-driven psoriasis is critically dependent on IL-36 signaling

**DOI:** 10.3389/fimmu.2023.1256133

**Published:** 2023-12-11

**Authors:** Berenice Fischer, Tanja Kübelbeck, Antonia Kolb, Julia Ringen, Ari Waisman, Miriam Wittmann, Susanne Karbach, Stephan Marcus Kölsch, Daniela Kramer

**Affiliations:** ^1^ Department of Dermatology, University Medical Center of the Johannes Gutenberg-University of Mainz, Mainz, Germany; ^2^ Center for Cardiology- Cardiology I, University Medical Center of the Johannes Gutenberg-University of Mainz, Mainz, Germany; ^3^ Center for Thrombosis and Hemostasis (CTH), University Medical Center of the Johannes Gutenberg-University of Mainz, Mainz, Germany; ^4^ Institute of Molecular Medicine, University Medical Center of the Johannes Gutenberg-University of Mainz, Mainz, Germany; ^5^ Research Center for Immunotherapy, University Medical Center of the Johannes Gutenberg-University of Mainz, Mainz, Germany; ^6^ German Center for Cardiovascular Research (DZHK) – Partner Site Rhine-Main, University Medical Center of the Johannes Gutenberg-University Mainz, Mainz, Germany; ^7^ Boehringer Ingelheim Pharma GmbH & Co. KG, Medical Affairs, Ingelheim am Rhein, Germany

**Keywords:** anti-IL36R, psoriasis, IL-17A, keratinocytes, imiquimod, systemic inflammation, spesolimab

## Abstract

Plaque psoriasis is an autoinflammatory and autoimmune skin disease, affecting 1-3% of the population worldwide. Previously, high levels of IL-36 family cytokines were found in psoriatic skin lesions, thereby contributing to keratinocyte hyperproliferation and infiltration of immune cells such as neutrophils. While treatment with anti-IL36 receptor (IL36R) antibodies was recently approved for generalized pustular psoriasis (GPP), it remains unclear, if targeting the IL36R might also inhibit plaque psoriasis. Here we show that antibody-mediated inhibition of IL36R is sufficient to suppress imiquimod-induced psoriasis-like skin inflammation and represses the disease’s development in a model that depends on IL-17A overexpression in the skin. Importantly, treatment with anti-IL36R antibodies inhibited skin inflammation and attenuated psoriasis-associated, systemic inflammation. This is possibly due to a widespread effect of IL36R inhibition, which not only suppresses pro-inflammatory gene expression in keratinocytes, but also the activation of other immune cells such as T-cells or dendritic cells. In conclusion, we propose that inhibition of the IL-36 signaling pathway might constitute an attractive, alternative approach for treating IL-17A-driven psoriasis and psoriasis-linked comorbidities.

## 1 Introduction

There are five main types of psoriasis – plaque, guttate, inverse, pustular, and erythrodermic psoriasis. In general, psoriasis is an autoimmune and autoinflammatory skin disease characterized by parakeratosis, acanthosis, and a significant infiltration of various immune cells, especially IL-17-producing cells, neutrophils, and pro-inflammatory monocytes (1). Psoriasis vulgaris or plaque psoriasis is the most prevalent subtype of this skin disease (about 90% of the cases), characterized by an overactive immune response, leading to a chronic expression of IL-23 and IL-17A cytokines. While the causes of psoriasis are still not fully understood, it has become clear that psoriasis is not only a skin condition but also influences the development of several comorbidities such as psoriatic arthritis, metabolic syndrome, and cardiovascular disease (2). Why and which patients develop comorbidities and how these can be effectively treated, are the focus of ongoing investigations. Thus, there is still a medical need for new anti-psoriatic therapy approaches that also effectively inhibit psoriasis-associated comorbidities.

Besides IL-17A and IL-23, two members of the IL-1 superfamily, IL-36α and IL-36γ, were found to be overexpressed in the epidermal compartment of psoriasis vulgaris skin lesions (3, 4). Therefore, IL-36 signaling constitutes an attractive alternative pathway that could be targeted for the treatment of plaque psoriasis. IL-36 belongs to the IL-1 family, consisting of three activating isoforms (IL-36α, IL-36β, IL-36γ) and one antagonist (IL36Ra) (5). For activation or repression of IL-36 signaling, all IL-36 cytokines need to undergo N-terminally cleavage by endogenous cathepsins, which are neutrophil- or pathogen-derived proteases (6–9). Subsequently, activated IL-36 agonists bind to the IL-36 receptor leading to the recruitment of the IL-1RAcP adaptor molecule and subsequent downstream activation of MyD88, NF-κB, and STAT3, thereby triggering pro-inflammatory target gene expression (5, 10, 11). IL-36 cytokines, as well as the IL-36 receptor, are widely expressed in various murine and human cell types, such as keratinocytes, endothelial cells, dendritic cells (DCs), and CD4^+^ T-cells (12–14). IL-36 stimulation of keratinocytes, for example, induces the expression of chemokines such as *Cxcl2*, *Cxcl5*, *Ccl2*, and *Ccl20*, triggering a massive infiltration of neutrophils, monocytes, and T-cells into the skin (11). Moreover, in CD4^+^ T-cells, IL-36 treatment induces the proliferation of naïve T-cells and polarization to T helper 1 (Th1) cells, whereas IL-36-stimulated DCs promote T-cell priming (12, 15, 16). Further effects of IL-36 stimulation on the activation and function of endothelial cells, monocytes, CD8^+^ T-cells, and γδ T-cells have also been reported (16–18). Hence, IL-36 signaling seems to be an important signaling pathway that - depending on the initial trigger - can act on multiple cell types, thereby eliciting innate but also adaptive immune responses.

Originally, the prominent role of IL-36 cytokines in psoriasis was noted in pustular psoriasis, particularly in GPP. This rare and often severe dermatosis, marked by sterile pustules, is characterized by overactive IL-36 signaling due to loss-of-function mutations in the IL-36 receptor antagonist *IL36RA* (19). Confirming this pathomechanism, an antibody targeting the IL-36 receptor (IL36R) has recently been successfully developed and approved for the treatment of GPP (20). Whether inhibition of IL-36 signaling could also constitute an attractive approach for the treatment of plaque psoriasis, remains to be investigated though. Interestingly, global and keratinocyte-specific deletions of IL36R are sufficient to prevent experimentally induced psoriasis in mice (21–23). IL-36 has been shown to induce IL-23, and importantly IL-17 and IL-36 cytokines can induce each other, thereby amplifying pro-inflammatory gene expression in keratinocytes (24–26). Thus inhibition of the IL-36 signaling pathway might potentially also inhibit IL-17A-driven inflammation in plaque psoriasis. Accordingly, it was previously shown that the application of an anti-IL-36R antibody partially prevents imiquimod (IMQ)-, IL-23, or IL-36-mediated skin inflammation in mice. However, it is not clear if treatment with an anti-IL36R antibody can suppress psoriasis-like skin inflammation once it is already established.

To clarify, if inhibition of IL-36 signaling can block IL-17A-driven plaque psoriasis, especially when inflammation has already been induced, we investigated the effects of systemic anti-IL36R antibody application in two different mouse models. Effects of an anti-IL-36R antibody treatment were investigated either after establishment of IMQ-induced psoriasis or in keratinocyte-specific IL-17A overexpressing mice (K14-IL17A^ind^ mice), harboring a heterogeneous deletion of the psoriasis-promoting factor IκBζ (*Nfkbiz*). Using both mouse models, we could show that inhibition of the IL-36 pathway effectively suppressed the recruitment of neutrophils, monocytes, T-cells, and dendritic cells to the affected skin. Moreover, keratinocyte hyperproliferation and psoriasis-associated, pro-inflammatory gene expression were effectively blocked in the skin of anti-IL36R-treated mice. We observed a widespread effect of anti-IL36R treatment, which affected local keratinocyte responses and the activation of other immune cells, leading to an inhibition of psoriasis-associated systemic inflammation. Thus, our data imply that inhibition of IL-36 signaling constitutes an attractive, alternative approach for the treatment of IL-17A-driven plaque psoriasis, which may also effectively inhibit psoriasis-associated comorbidities.

## 2 Results

### 2.1 IL-36 signaling is upregulated in human psoriasis vulgaris, and mouse models of psoriasis

**Figure 1:**
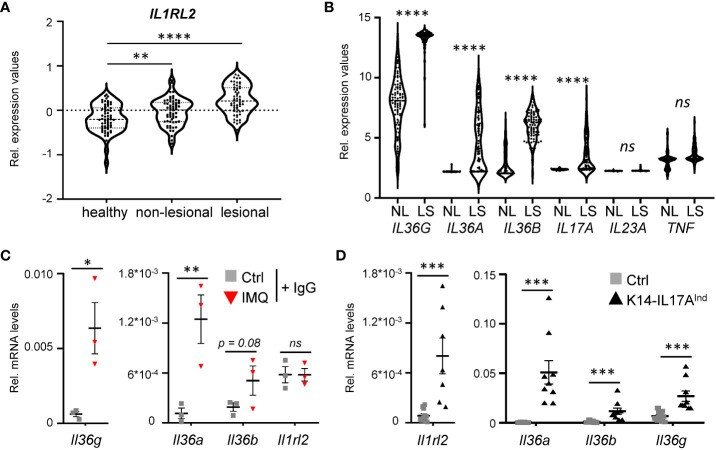
IL-36 signaling is upregulated in psoriatic skin lesions of mice and psoriasis patients. **(A)** mRNA expression levels of the IL36 receptor *(IL36R/IL1RL2*) in skin samples of 64 healthy individuals and in non-lesional and lesional samples from 58 psoriasis vulgaris patients (GDS4602). Significance was calculated using a 2-tailed Student’s t-test: ** p < 0.01**** < 0.0001. **(B)** mRNA expression levels of psoriasis-associated cytokines in lesional and non-lesional skin of 85 patients (GDS4600). Significance was calculated using a one-way ANOVA test. ns = not significant, **** p < 0.0001. **(C, D)** Gene expression of IL-36 cytokines and *Il1rl2* in 7 days IMQ-treated skin **(C)** or lesions of K14-IL17A^ind^ mice **(D)**. Shown are the relative mRNA levels normalized to *β-Actin*. Significance was calculated using a 2-tailed Student’s t-test: * p < 0.05, ** p < 0.01, *** < 0.001, *ns*, not significant. Shown is the mean ± SEM of n = 3 **(C)** or n = 9-11 **(D)** animals.

As a starting point of the project, we analyzed expression levels of the IL-36 receptor and IL-36 cytokines in human psoriasis vulgaris samples. As already reported before, IL-36 receptor expression (encoded by *IL1RL2*) (**Figure 1A**), as well as the expression of *IL36A*, *IL36B*, and *IL36G* are significantly upregulated in psoriasis vulgaris skin lesions, compared to healthy and non-lesional skin samples (**Figure 1B**) (27). Interestingly, when we re-analyzed the expression levels of known psoriasis-associated cytokines in previously published data sets, comparing gene expression of skin lesions to non-lesional controls derived from the same patient, *IL36A*, *IL36B*, and *IL36G* displayed the most significant increase in expression compared to conventional cytokines such as *IL17A*, *IL23A* or *TNF* (**Figure 1B**) (28, 29). Thus, IL-36 is a valid target for the treatment of plaque psoriasis, since IL-36 signaling is upregulated in plaque psoriasis, and its upregulation is even more pronounced than IL-17A and IL-22, which have already been successfully used as targets for the treatment of psoriasis vulgaris using neutralizing antibodies.

Furthermore, we detected a similar upregulation of the IL-36 signaling pathway in psoriasis-like skin lesions of mice that either develop psoriasis upon IMQ treatment (**Figure 1C**), or in psoriatic mice that overexpress IL-17A in the epidermis (**Figure 1D**). Thus, IL-36 signaling is also upregulated in two mouse models of IL-17A-driven psoriasis, which can subsequently be utilized to investigate anti-IL36R antibody treatment in IL-17A-driven psoriasis.

### 2.2 Keratinocyte hyperproliferation and immune cell infiltration are suppressed by anti-IL36R treatment in pre-established, IMQ-induced psoriasis

**Figure 2:**
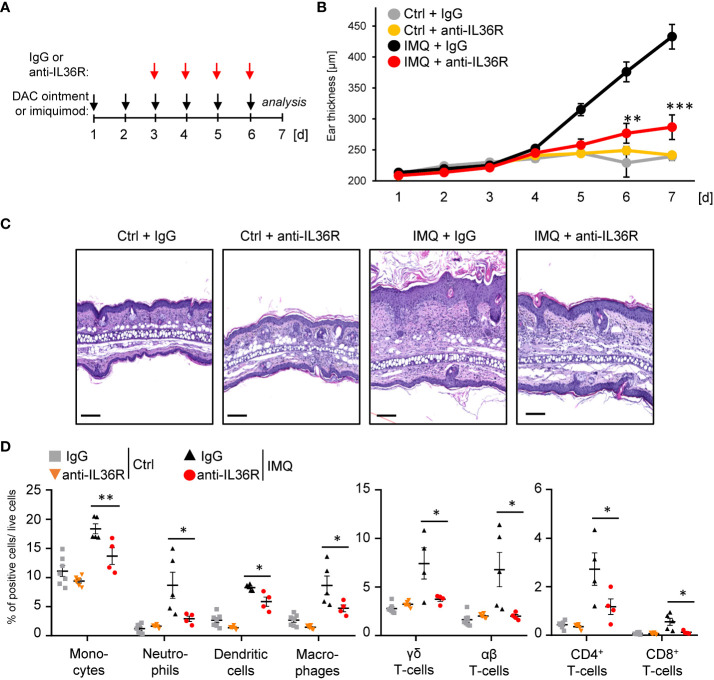
Anti-IL36R treatment suppresses keratinocyte hyperproliferation and infiltration of immune cells in IMQ-induced psoriasis-like skin inflammation. **(A)** Treatment scheme. Antibody treatment was started 2 days after IMQ treatment. **(B)** Ear thickness during the course of the experiment (n = 14, except for Ctrl + anti-IL36R n = 6). **(C)** H&E staining of ears from the treated mice at the endpoint at day 7. Scale: 100 µm. **(D)** Flow cytometry analysis of skin-infiltrating immune cells from treated animals at day 7 (n = 3 - 5 animals per group). Shown is the relative percentage of positive cells, after pre-gating on viable cells. Significance was calculated using a 2-tailed Student’s t-test: * p < 0.05, ** p < 0.01, *** < 0.001. Shown is the mean ± SEM.

Previously, it was shown that inhibition of IL-36 signaling by treatment with an anti-IL36R antibody or the IL-36RA antagonist partially prevents the development of IMQ- or IL-36-mediated psoriasis in mice (30–32). However, it remains unclear, if IMQ-induced psoriasis can be treated with an anti-IL36R antibody, once the skin inflammation has already developed. To test this, we pre-treated the ears of wildtype mice for 2 days with IMQ-containing Aldara® cream to induce psoriasis-like skin lesions. At this time point, keratinocyte hyperproliferation and gene expression of psoriasis-associated cytokines and chemokines were already detectable (**Supplementary Figures 1A, B**). Subsequently, from day 3 on, besides the topical IMQ treatment, mice received intraperitoneal (*i.p.*) injections of either IgG control or anti-IL-36R antibody (**Figure 2A**), which was able to inhibit IL-36-driven gene expression in keratinocytes *in vitro* (**Supplementary Figure 1C**). On day 7, mice were sacrificed and subsequently analyzed. Interestingly, systemic application of an anti-IL36R antibody was not only able to effectively inhibit ear swelling, but also significantly reduced keratinocyte hyperproliferation and infiltration of neutrophils, monocytes, dendritic cells, and T-cells into the ears of IMQ-treated mice (**Figures 2B-D**). Moreover, blockade of the IL-36 signaling pathway also suppressed systemic inflammation marked by elevated blood counts of neutrophils, monocytes, and dendritic cells in IMQ-treated mice (**Supplementary Figure 1D**).

### 2.3 Anti-IL36R treatment suppresses the expression of psoriasis-associated cytokines and chemokines in IMQ-treated mice

**Figure 3:**
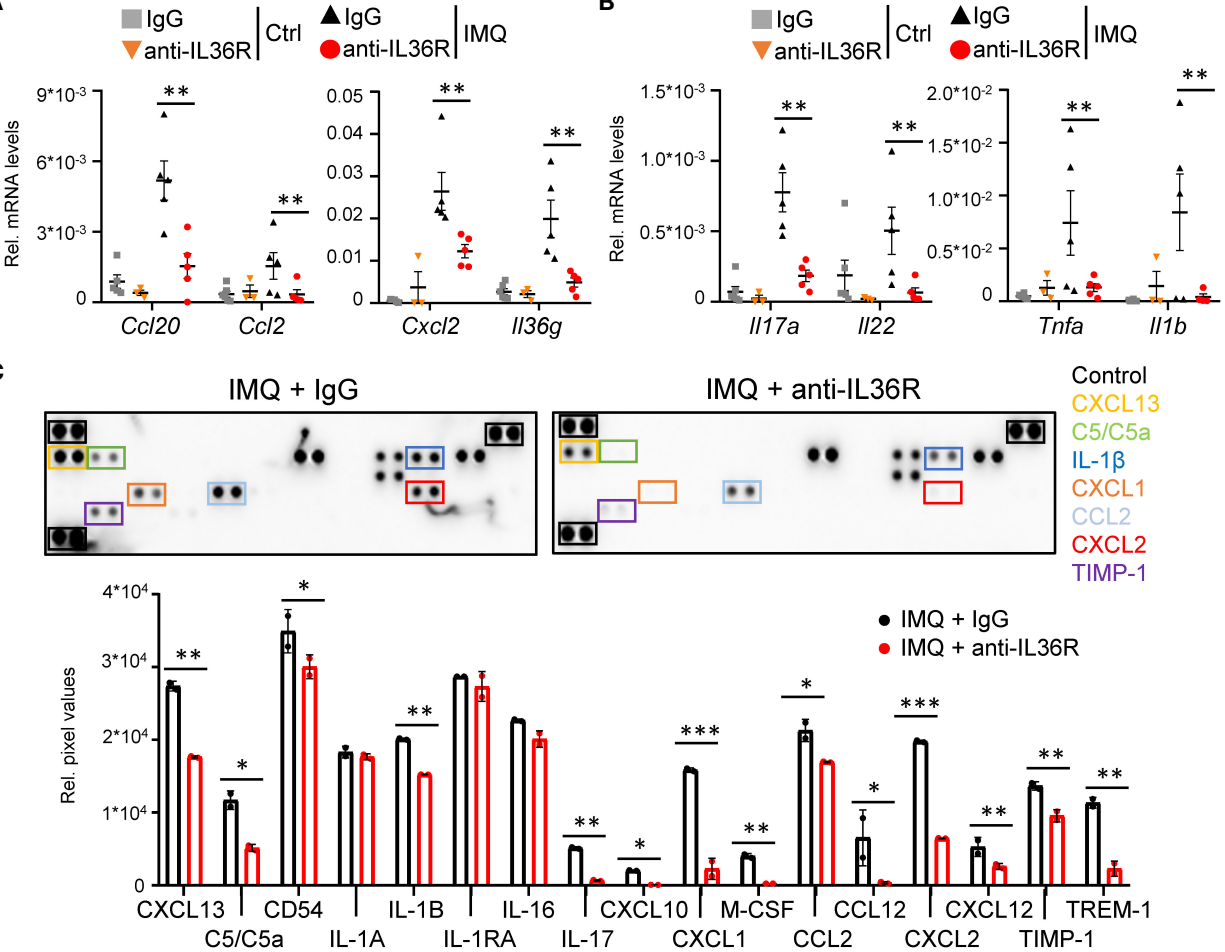
Anti-IL36R treatment suppresses pro-inflammatory cytokine and chemokine expression in IMQ-induced psoriasis-like skin inflammation. **(A, B)** Gene expression analysis of the skin from Ctrl or IMQ-treated animals that received IgG or anti-IL36R treatment. Shown is the relative gene expression of keratinocyte-derived cytokines and chemokines **(A)**, as well as of psoriasis-associated cytokines deriving from T-cells and myeloid cells **(B)**, normalized to *β-Actin*. n = 3 - 6. Shown is the mean ± SEM. **(C)** Protein levels of cytokines and chemokines from ears of IMQ + IgG and IMQ + anti-IL36R-treated animals. Shown are the immunoblot analysis and its evaluation normalized to the reference controls and depicted as relative pixel values. Samples were derived from 3 animals per group and pooled before analysis. Significance was calculated using a 2-tailed Student’s t-test: *p < 0.05, **p < 0.01, and ***p < 0.001.

Next, we wanted to understand, which molecular signaling pathways are affected by anti-IL36R treatment in IMQ-treated mice. As expected, suppression of IL 36 signaling did not only affect the expression of mostly keratinocyte-derived chemokines (*Ccl20*, *Ccl2*, *Cxcl2*) and cytokines (*Il36g*) (**Figure 3A**) but also effectively reduced the expression of psoriasis-associated cytokines produced by T-cells (*Il17a, Il22, Tnf*) and myeloid cells (*Il1b*) (**Figure 3B**, **Supplementary Figures 2A-C**). Importantly, the same chemokines and cytokines (e.g. CXCL2, CCL2, IL-1β, and IL-17) were also effectively downregulated on the protein level in IMQ- and anti-IL36R-treated animals compared to IMQ- and IgG-treated mice (**Figure 3C**, **Supplementary Figure 2D**). Thus, systemic anti-IL36R treatment was able to broadly inhibit psoriasis-like skin inflammation in IMQ-treated mice.

### 2.4 Anti-IL36R antibody treatment inhibits the development of psoriasis-like skin lesions in keratinocyte-specific IL-17A-overexpressing mice

**Figure 4:**
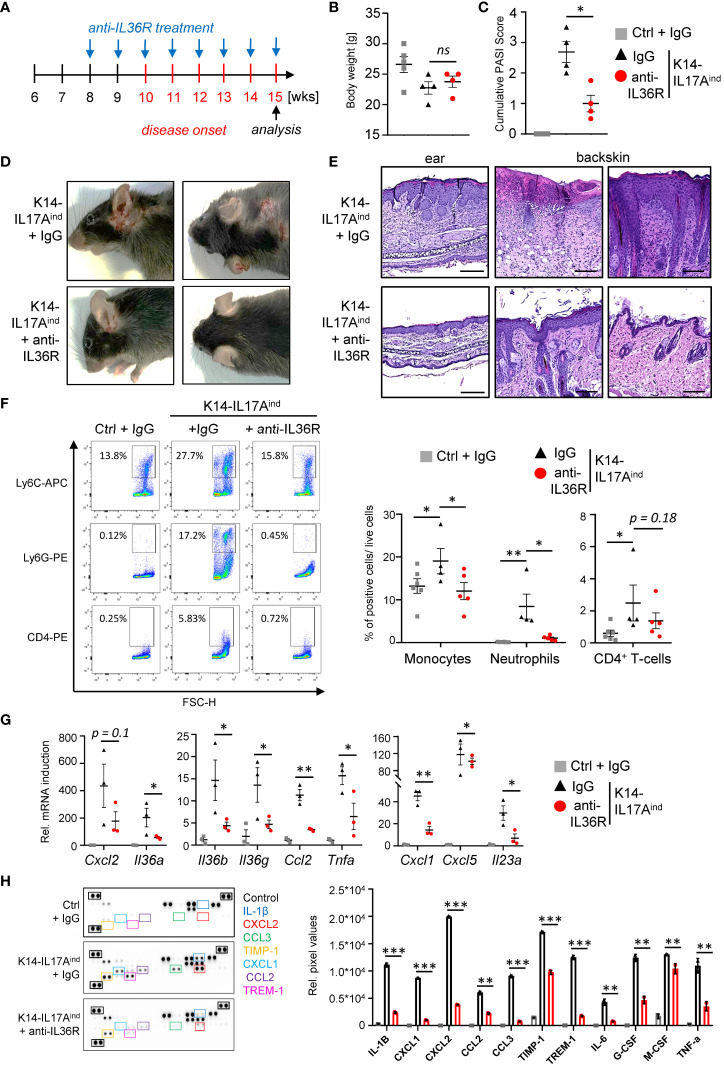
Anti-IL36R treatment suppresses skin lesion development and immune cell infiltration in IL-17A-induced psoriasis *in vivo*. **(A)** Treatment scheme. **(A, C)** Body Weight **(B)** and PASI Score **(C)** of the animals at the endpoint (15–20 weeks old). **(D)** Pictures of the animals at endpoint. **(E)** H&E staining of the ears and skin lesions at the endpoint. Scale: 100 µm (ears) and 200 µm (back skin). **(F)** Flow cytometry analysis of skin-infiltrating immune cells from treated animals at endpoint (n = 4 - 6 animals per group). Shown is the relative percentage of positive cells, after pre-gating on viable cells. **(G)** Gene expression analysis of the skin of IgG- or anti-IL36R-treated K14-IL17A^ind^ mice. Relative gene expression was normalized to *Rpl37A*. Shown is the gene expression from one treatment group. **(H)** Protein levels from lesional skin of IgG- and anti-IL36R-treated K14-IL17A^ind^ mice. n = 3 animals per group. Significance was calculated using a 2-tailed Student’s t-test: * p < 0.05, ** p < 0.01, *** < 0.001, ns, not significant. Shown is the mean ± SEM.

Based on our findings using the IMQ mouse model, we next investigated the effects of anti-IL36R antibody treatment in a genetic mouse model of IL-17A-driven psoriasis. K14-IL17A^ind^ mice develop psoriasis-like skin lesions due to constitutive, keratinocyte-derived overexpression of IL-17A (33). Of note, we utilized K14-IL17A^ind^ mice harboring a heterozygous deletion of *Nfkbiz*, a well-known key factor of transcriptional responses in psoriasis (11, 34). Whereas K14-IL17A^ind^ mice harboring wildtype *Nfkbiz* expression show severe systemic inflammation and skin lesions all over the whole body at young age (33, 35), keratinocyte-specific, heterozygous depletion of *Nfkbiz* in these mice renders the mice healthy until an age of 10–11 weeks, when the mice start to develop psoriasis-like skin lesions at the ears and back (34). Due to the temporal appearance and extent of psoriatic skin disease in these mice, we therefore decided to use this mouse model. Mice received *i.p.* injections with IgG control or anti-IL36R antibody once a week, starting at an age of 8 weeks (**Figure 4A**). Development of psoriasis was scored using a cumulative *Psoriasis Area and Severity Index* (PASI) score describing the lesion size and the severity of scaling and erythema at the sites of the lesions (34). At around weeks 15-20, when at least one mouse of a treatment group reached a PASI score of 3-4, all mice were sacrificed and analyzed. Although IgG- or anti-IL36R antibody-treated K14-IL17A^ind^ mice overexpressed comparable levels of IL-17A (**Supplementary Figure 3A**) and showed comparable body weights (**Figure 4B**), blockade of the IL-36 signaling pathway was sufficient to partially inhibit the development of psoriatic lesions in these mice (**Figures 4C, D**). Likewise, keratinocyte hyperproliferation (**Figure 4E**) and the relative infiltration of neutrophils and monocytes into the skin of K14-IL17A^ind^ mice were also reduced in mice that received anti-IL-36R antibody injections (**Figure 4F**).

Molecular analysis of the skin of IgG- and anti-IL36R-treated K14-IL17A^ind^ mice revealed effective suppression of psoriasis-associated cytokines (*Il36a, Il36g, Il36b, Il23a*, and *Tnf*), chemokines (*Cxcl1, Cxcl5*, and *Ccl2*) (**Figure 4G**) and antimicrobial-peptides (such as *Defb4*) on mRNA level (**Supplementary Figure 3B**). Moreover, several cytokines and chemokines, such as IL-1β, IL-6, TNF, CXCL2, and CCL2 were efficiently suppressed on protein level in the skin of anti-IL36R-treated K14-IL17A^ind^ mice, compared to the IgG-treated control mice (**Figure 4H**). Thus, application of anti-IL36R antibodies effectively suppressed the development of IL-17A-driven psoriasis-like skin inflammation in these mice.

### 2.5 Inhibition of IL-36 signaling attenuates psoriasis-associated systemic inflammation in K14-IL17A^ind^ mice

**Figure 5:**
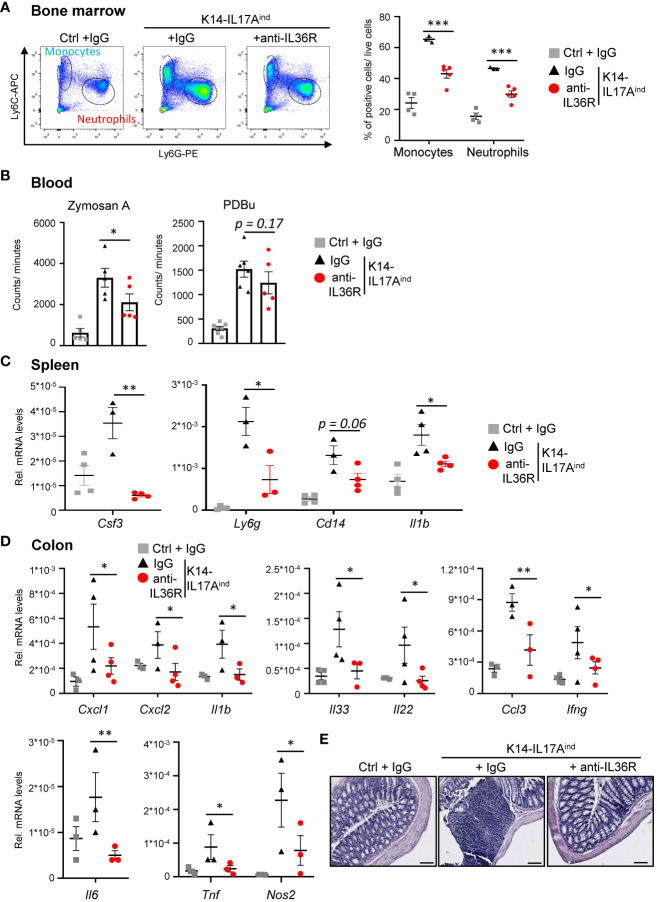
Anti-IL36R treatment inhibits systemic inflammation in K14-IL17A^ind^ mice. **(A)** Flow cytometry analysis of the bone marrow from Ctrl mice or K14-IL17A^ind^ mice that either received IgG or anti-IL36R treatment (n = 2-3 animals per group). The relative percentage of positive cells is shown after pre-gating on viable cells. **(B)** Oxidative burst was measured in venous blood of control or K14-IL17A^ind^ mice by analyzing the formation of reactive oxygen and nitrogen species. Blood cells were either restimulated with phorbol-12,13-dibutyrate (PDBu) or with Zymosan A. After addition of the 8-amino-5-chloro-7-phenylpyridol-(3,4-d)pyridazine-1,4-(2H,3H)-dione sodium salt (L-012), the oxidative burst was detected by the created chemiluminescence using a SparkTM Multimode Microplate Reader. n = 4-5. C + D. Gene expression analysis of the spleen **(C)** and colon **(D)** from treated mice. Shown is the relative gene expression normalized to *β-Actin* **(C)** or *Rpl37A* **(D)** for n = 3-4 animals per group. Significance was calculated using a 2-tailed Student’s t-test: * p < 0.05, ** p < 0.01, *** p< 0.001. Shown is the mean ± SEM. **(E)** H&E staining of the colon at the endpoint. Scale: 100 µm.

K14-IL17A^ind^ mice do not only suffer from psoriasis-like skin lesions but also develop psoriasis-associated systemic inflammation, similar to psoriasis-related comorbidities in humans. Therefore, we investigated if the application of an anti-IL36R antibody was also able to inhibit the development of systemic inflammation in K14-IL17A^ind^ mice. The first signs of systemic inflammation can be detected by increased granulopoiesis and myelopoiesis in the bone marrow. Next, these myeloid cells are released into the bloodstream, become overactive, and elicit more reactive oxygen species (ROS), which subsequently leads to vessel damage and the development of cardiovascular disease in the long run (35). Subsequently, systemic inflammation in several organs develops, which is marked by an increased expression of several cytokines and chemokines. This process may be due to, or triggered by, this systemic increase in myeloid cells and their activation and contributes to the development of psoriasis-associated comorbidities. Congruent with the less severe psoriatic skin lesions in anti-IL36R-treated K14-IL17A^ind^ mice, we also detected significantly fewer monocytes and neutrophils being generated in the bone marrow (**Figure 5A**). Reactive oxygen species in the blood were found to be significantly increased in psoriatic mice as compared to healthy controls. Upon anti-IL36R treatment, the increase in ROS formation was still observed but was no longer significant (**Figure 5B**). Furthermore, whereas IgG-treated K14-IL17A^ind^ mice showed signs of inflammation in the spleen and colon, as well as an increased expression of pro-inflammatory cytokines (such as *Csf3, Il1b, Il6*, and *Tnf*), treatment with an anti-IL36R antibody remarkedly inhibited systemic signs of inflammation in the organs of these mice (**Figures 5C-E**). This suggests, that in addition to suppression of the psoriatic skin disease, anti-IL36R antibodies could also inhibit psoriasis-associated systemic inflammation in K14-IL17A^ind^ mice.

### 2.6 Acute treatment with anti-IL36R antibodies partially resolves inflammation in K14-IL17A^ind^ mice

**Figure 6:**
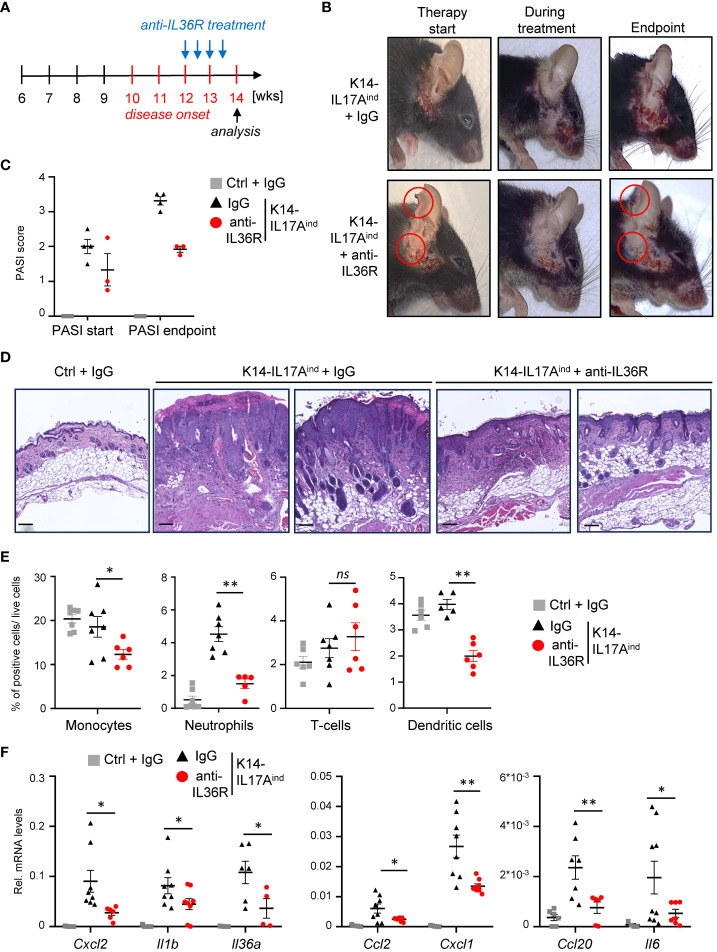
Acute suppression of psoriasis-associated inflammation in K14-IL17A^ind^ mice upon anti-IL36R therapy. **(A)** Treatment scheme. **(B)** Pictures of an IgG or anti-IL36R-treated animal at the starting point of the treatment, during the treatment and at the endpoint. **(C)** PASI Score of the mice before treatment and at the endpoint. **(D)** H&E staining of the affected skin areas at the endpoint. Scale: 100 µm. **(E)** Flow cytometry analysis of skin-infiltrating immune cells from treated animals at endpoint (n = 3 - 5 animals per group). Shown is the relative percentage of positive cells, after pre-gating on viable cells. **(F)** Gene expression of the skin from IgG or anti-IL36R-treated K14-IL17A^ind^ mice at the endpoint. Relative gene expression was normalized to *Rpl37A*. Significance was calculated using a 2-tailed Student’s t-test: * p < 0.05, ** p < 0.01, ns, not significant. Shown is the mean ± SEM.

Next, we wanted to investigate if anti-IL36R antibody treatment is also able to inhibit and resolve psoriasis in K14-IL17A^ind^ mice once the psoriatic lesions already developed. For this purpose, we waited until approximately week 12, when the mice were scored with a PASI score of 1-2 and already displayed psoriatic lesions at the head and neck. Subsequently, we treated the mice two times a week for 2-3 weeks with either IgG as control or anti-IL36R antibody, until at least one animal of the control or experimental group reached a PASI score of approximately 4. At this time point (around 14-15 weeks of age), all mice were sacrificed and analyzed (**Figure 6A**). Interestingly, this short-term treatment with anti-IL36R antibody was sufficient to significantly diminish the lesion size, keratinocyte hyperproliferation, and infiltration of neutrophils, monocytes, and dendritic cells into the psoriatic skin lesions of K14-IL17A^ind^ mice (**Figures 6B-E**). This was due to a significant repression of psoriasis-associated cytokine and chemokine expression in the previously affected lesional skin of anti-IL36R-treated animals, compared to IgG-treated control animals (**Figure 6F**), and to non-lesional skin samples of the same, anti-IL36R-treated mice (**Supplementary Figure 4A**). Furthermore, whereas IgG-treated K14-IL17A^ind^ mice also displayed psoriasis-associated systemic inflammation, marked by granulopoiesis, myelopoiesis, and an elevated expression of inflammation markers in the spleen, mice that received a short-term treatment with anti-IL36R antibodies displayed a reduction in these markers of systemic inflammation (**Supplementary Figures 4B, C**).

In conclusion, anti-IL36R treatment is not only able to prevent the development of IL17A-driven psoriasis but also to inhibit and partially resolve psoriatic lesions and associated psoriasis-associated systemic inflammation.

## 3 Discussion

An antibody targeting the IL-36 receptor has recently been approved for the treatment of GPP, a subtype of psoriasis (19). While this treatment is very effective in GPP, it remains an open question if an anti-IL36R antibody might also be beneficial for the treatment of IL-17A-dependent plaque psoriasis. Moreover, inhibition of IL-36 signaling in psoriasis might also be advantageous for the effective treatment of psoriasis-associated comorbidities. Here, we investigated the consequences of an anti-IL36R treatment in two mouse models of IL-17A-driven psoriasis: in IMQ-treated mice and in mice that overexpress IL-17A in the epidermis, thus developing IL-17A-depending psoriatic lesions. We found that systemic anti-IL36R treatment, given after the development of IMQ-induced psoriasis-like skin lesions, is able to inhibit keratinocyte hyperproliferation and immune cell infiltration into IMQ-treated skin. Furthermore, it was sufficient to repress skin-derived, pro-inflammatory cytokine and chemokine expression. Our findings are in agreement with previous studies, in which a global and keratinocyte-specific knockout of the IL-36 receptor (21, 23), or anti-IL-36R treatment (30, 36) was sufficient to effectively prevent IMQ-induced psoriasis-like skin inflammation. Limitations of the previous studies were, on the one hand, the experimental setup, as well as secondly, the IMQ model itself. In the previous studies, IL36R knockout or inhibition of IL-36 signaling was already present before the application of IMQ was started, thereby only showing that blockade of the IL-36 pathway can prevent IMQ-mediated skin inflammation (21, 23, 30, 36). We here add to these data by showing that anti-IL36R antibody treatment is also able to attenuate already fully established IMQ-mediated skin inflammation. Thus, anti-IL36R treatment efficiently blocks IMQ-mediated psoriasis-like skin disease in mice, similar to what has previously been shown for neutralizing antibodies against IL-17 or IL-23 (37, 38).

The other limitation of the previous studies derives from the IMQ mouse model itself. While in patients, triggers for the induction of psoriatic lesions are multifactorial but seem to derive from “stressed” keratinocytes (39), IMQ constitutes a TLR7/8 agonist, that activates skin-resident immune cells in the first instance. Thus, although IMQ is still the standard model for testing anti-psoriatic drugs, it remains to be seen if it is the best pre-clinical model available (40). Furthermore, it has recently been shown that IMQ fails to induce a pure psoriasis response in humans but instead causes an IL-17-dominated contact dermatitis (41). Therefore, we decided to additionally investigate the consequences of an anti-IL36R treatment in a genetically driven psoriasis mouse model. K14-IL17A^ind^ *Nfkbiz* het mice (referred to as K14-IL17A^ind^) which chronically overexpress IL-17A in the epidermal compartment, in combination with a heterozygous depletion of *Nfkbiz* in keratinocytes. While these mice look completely healthy at birth, they develop psoriatic skin lesions at the head and back in adulthood (34). The disease slowly progresses, and subsequently also triggers the development of psoriasis-associated comorbidities, thus much better reflecting the human psoriasis pathology (34, 35). Interestingly, while IgG control-treated K14-IL17A^ind^ mice displayed psoriasis-like skin lesions starting at an age of 11 weeks, mice that were treated with anti-IL36R antibodies in parallel developed significantly fewer and less severe lesions, although exhibiting comparable levels of IL-17A overexpression. Moreover, inhibition of IL-36 signaling was able to effectively inhibit particularly the infiltration of monocytes and neutrophils into the skin lesions, as well as the expression of known psoriasis-associated cytokines and chemokines. Importantly, using this mouse model, we could also show that short term treatment using anti-IL36R antibodies was sufficient to successfully inhibit psoriasis-associated inflammation once it has already been established.

Previously, it was shown that IL-23, IL-17, and IL-36 expression are closely interconnected with each other in keratinocytes and that IL-36, downstream of IL-23 and IL-17, constitutes a critical effector molecule of IL-23 and IL-17 responses in psoriasis (42–44). Thus, IL-17A secretion by T-cells does not only induce the expression of IL-36α and IL-36γ in various cell types but also synergistically induce the expression of various cytokines and chemokines driving the progression of psoriasis (14, 24). Vice versa, IL-36γ expression in psoriatic lesions is one of the earliest genes which become repressed upon treatment with anti-IL17 antibodies (14). In agreement with these findings, we detected a strong downregulation of several IL-23 and IL-17 target genes (such as *Il36g*, *Defb4*, or *S100a9*) in the skin of anti-IL36R-treated mice. These genes have previously been shown to be important markers for effective therapeutic responses in human psoriasis vulgaris patients, treated with anti-IL17 or anti-IL23 antibodies (45, 46). Thus, since anti-IL36R treatment downregulates similar genes as anti-IL17 or anti-IL23 therapy does, blockade of the IL-36 signaling pathway might be also similar effective as other biologicals for the treatment of psoriasis vulgaris patients.

IgG-control-treated K14-IL17A^ind^ mice also suffered from psoriasis-associated systemic inflammation, which is characterized by an elevated granulopoiesis, myelopoiesis, inflamed spleen, and colon. There were no signs of systemic inflammation detectable in the simultaneously aged and anti-IL36R-treated K14-IL17A^ind^ mice. We suggest that these effects derive from the widespread anti-inflammatory effect of anti-IL-36 antibody treatment, as previous studies revealed that not only keratinocytes but also endothelial cells, fibroblasts, T-cells, and dendritic cells respond to IL-36 (13, 16, 47). Our findings have major therapeutic implications, since antibodies against IL36R might not only be important for the direct treatment of the psoriatic skin but also for psoriasis-associated comorbidities, which impact life expectancy. Previous studies showed that IL-36 overexpression contributes to the development of arthritis (4), metabolic disorders (48), cardiovascular diseases (49), and inflammatory bowel disease (50). Future approaches should therefore investigate to what extent IL-36 overactivation contributes to the development of psoriasis-associated comorbidities, and if anti-IL36R antibodies, alone or in combination with other anti-psoriatic drugs, are helpful in the treatment of these comorbidities.

In summary, our pre-clinical study showed that inhibition of the IL-36 signaling pathway constitutes an attractive approach for the treatment of IL-17A-driven psoriasis vulgaris. As it is still not clear which class of neutralizing antibodies are the most efficient therapy for the whole psoriatic disease spectrum and with regard to long-term disease trajectories, anti-IL36R antibodies might constitute an attractive alternative with unique properties targeting both skin and systemic symptoms.

## 4 Materials and methods

### 4.1 Mice

All animal experiments have been approved by the local animal ethics committee (Landesuntersuchungsamt Rheinland-Pfalz). All experiments were conducted in accordance with German law and guidelines for animal care. For the anti-IL36R treatment of IMQ-induced psoriasis, both ears of male C57BL/6NCrl wild-type mice (8-10 weeks old, purchased from Charles River Laboratories) were treated for 6 consecutive days with Aldara® cream (5% IMQ, 3M Pharmaceuticals), while control mice received diluted basis cream (DAC). After 2 days of treatment with DAC or IMQ, mice additionally received intraperitoneal (*i.p.)* injections of either 750 µg IgG or anti-IL36R antibodies (both from Boehringer Ingelheim) from the third day of IMQ treatment onwards. Ear thickness was daily measured using a precise caliper (IP67/C110T, Kroeplin). On day 7, mice were sacrificed and analyzed.

To study the effect of anti-IL36R treatment in IL-17A-driven psoriasis, both female and male K14-IL17A^ind^ *Nfkbiz* ^fl/+^ mice were utilized (referred to as K14-IL17A^ind^), which develop skin lesions starting at an age around 11 weeks old. These mice were generated by crossing homozygous KRT14-Cre mice (B6N.Cg-Tg(KRT14-cre)1Amc/J) from Jackson Laboratories (Stock 018964) to homozygous IL17A^ind^ *Nfkbiz* ^fl/fl^ (B6.Gt(ROSA)26Sor^tm3(CAG-Il17a)Awai^; Nfkbiz^tm1.1Muta^/Tarc) mice. Starting at an age of 8 weeks, K14-IL17A^ind^ mice were intraperitoneally injected with 500 µg IgG or anti-IL36R antibody once a week. As a control, IL17A^ind^ *Nfkbiz* ^fl/+^ mice (referred to as Ctrl) of the same age and gender were treated with IgG antibodies. Mice were scored with the cumulative PASI Score describing the degree of scaling, erythema, and percentage of the affected area (0 = no lesions, 1 = very mild, 2 = mild, 3 = intermediate, 4 = severe, and 5 = very severe). When at least one animal of the treatment group reached a PASI score of 3 - 5, at an age of 15 - 20 weeks, all mice of the same treatment group were sacrificed and analyzed in parallel. For the therapeutic approach, control and K14-IL17A^ind^ mice were treated with 500 µg IgG or anti-IL36R antibody twice a week for 3 to 4 weeks, starting as soon as a PASI score of 1-2 was reached. Sacrification and analysis were carried out after reaching the endpoint as described above.

### 4.2 Isolation and treatment of murine keratinocytes from tails

8- to 12-weeks-old wildtype mice were sacrificed and tails were wiped with 70% isopropanol. In order to isolate mKC, the skin was separated from the muscle and incubated overnight at 4°C in equal amounts of basal keratinocyte-SFM medium (Cat.17005042 Gibco, Thermo Fisher Scientific) and 5 U/mL dispase I solution (Cat.07913, StemCell). The next day, the epidermis was separated from the dermis and incubated for 15 min with 0.05% trypsin-EDTA solution (Cat.25300-054, Thermo Fisher Scientific) at room temperature (RT). Stopping the reaction with DMEM medium supplemented with 10% FCS, cells were carefully washed out and transferred to a collection tube using a 100 µm cell strainer. After centrifugation (1000 rpm, 5 min), the pellet was resuspended in complete keratinocyte-SFM medium (containing BPE and EGF), supplemented with 0.05 M CaCl_2_. Cells were seeded on collagen type I-coated 6-well plates (Cat.A1064401, Thermo Fisher Scientific). When the plates reached a confluency of 70-80% confluency, cells were starved overnight (SFM Medium without CaCl_2_ and supplements) and subsequently treated with 100 ng/mL IL-36α (Cat.555902, Biolegend) for 1.5 h, in the presence of either 50 µg/mL IgG or anti-IL36R antibody.

### 4.3 RNA extraction and gene expression analysis by quantitative PCR

Total RNA isolation and qPCR analysis were performed as described before (51). Briefly, 1 or 4 µg total RNA (from cell culture samples or tissue) was reverse transcribed into cDNA. Gene expression was quantified by real-time PCR (CFX384, BioRad) using self-designed primers (**Supplementary Table 1**) and GreenMasterMix (Cat.M3023, Genaxxon) using the following PCR protocol: 15 min initial denaturation at 95°C, 40 cycles of 95°C for 15 s and 60°C for 45 s. Relative mRNA levels were normalized to the reference gene *ActB* (keratinocytes, skin, spleen), *Rpl37a* (colon) using the 2^-ΔCt^ method.

### 4.4 ELISA

Quantitative levels of murine IL-17A were measured in skin and serum samples using the commercial ELISA MAX™ Deluxe Set Mouse IL-17A kit (Cat.432504, Biolegend). Measurement was performed using a microplate reader (Hidex Sense, Hidex) and concentration was determined based on four parameter logistic (4PL) regression.

### 4.5 Cytokine array

Secretion of cytokines was analyzed using the Proteome Profiler Mouse Cytokine Array Kit Panel A (Cat.ARY006, R&D systems) according to the manufacturer′s instructions. Skin samples were homogenized with C-tubes (Miltenyi) using the gentleMACS™ dissociator with protein lysis buffer containing 20 mM TRIS-HCl pH 7.5, 150 mM NaCl, 1% Triton X-100, 1 mM Na_2_EDTA, 1 mM EGTA, 1 mM β-glycerophosphate, 2 M urea and 1x protease inhibitor cocktail (Cat.4693124001, Sigma). After 10 min incubation on ice, samples were briefly sonicated (Bioruptor, Diagenode), centrifuged (1000 rpm, 5 min, 4°C), and the supernatant transferred into a new tube. Subsequently, the protein concentration was determined using the Qubit Protein Assay (Cat.Q33212, Thermo Fisher Scientific). Prior to analysis, input lysates were normalized to equal β-Actin levels by immunoblot analysis using an anti-β-Actin antibody (Cat.3700, Cell signaling). For each treatment group, equal amounts of samples deriving from 3 different animals were pooled and analyzed together. Membranes were developed using the Fusion FX imager (Peqlab) and relative cytokine expression levels were calculated as relative pixel values, normalized to the reference spots, using the dot blot analyzer (ImageJ).

### 4.6 Detection of reactive oxygen and nitrogen species in whole blood

The venous blood of the mice was taken by heart puncture and directly anti-coagulated with sodium citrate (0.1% v/v) to analyze the formation of reactive oxygen and nitrogen species (RONS) as previously described (52, 53). The blood was either incubated for 15 min with phorbol-12,13-dibutyrate (PDBu) or over 40 min with Zymosan A to induce oxidative burst of the whole blood. After the addition of the chemiluminescence dye 8-amino-5-chloro-7-phenylpyridol-(3,4-d)pyridazine-1,4-(2H,3H)-dione sodium salt (L-012), the created chemiluminescence directly correlating with the underlying ROS formation was measured, using a Spark™ Multimode Microplate Reader (Tecan Trading AG, Männedorf, Switzerland).

### 4.7 Flow cytometry

For analysis of infiltrating immune cell populations in skin samples, back skin or whole ears of approx. 1.5 x 1.5 cm was chopped and digested for 1.5 h at 37°C while shaking using 1 mL digestion mix containing 0.25 mg/mL Liberase TM (Cat.5401127001 Sigma), 100 µg/mL DNase I (Cat.11284932001 diluted in HBSS, Sigma), 0.5 mM CaCl_2_ and basal RPMI medium without supplements. Afterwards, skin samples were homogenized with C-tubes (Miltenyi) using the gentleMACS™ dissociator. The suspension was transferred to a 50 mL centrifuge tube using a 100 µm strainer to obtain a single-cell suspension and the digestion was stopped by adding 20 mL DMEM medium, supplemented with 10% FCS. After centrifugation (10 min at 450 x g) the cell pellet was washed in a FACS buffer containing 5% FCS and 1 mM EDTA. For preparation of bone marrow samples, cells were extracted from femur and tibia and washed in FACS buffer. Subsequently, prepared single-cell suspensions of skin and bone marrow were treated with TruStain FcX anti-mouse (Cat.101320, Biolegend) for 10 min on ice, washed and further surface-stained for 30 min on ice in the dark using the following antibodies: anti-CD3-APC (Cat.100236, clone 17A2, BioLegend), anti-CD4-PE (Cat.100408, clone GK1.5, BioLegend), anti-CD8a-APC (Cat.100712, clone 53-6.7, BioLegend), anti-Ly6G-PE (Cat.127607, clone 1A8, BioLegend), anti-Ly6C-APC (Cat.128016, clone HK1.4, BioLegend), anti-CD11c-APC (Cat.117310, clone N418, BioLegend), anti-F4/80-PE (Cat.123110, clone BM8, BioLegend), anti-αβTCR-PE (Cat.22155214, clone H57-597, Immunotools) and anti-γδ-APC (Cat.118115, clone GL3, BioLegend). Data was acquired on a LSR II flow cytometer (Becton Dickson) and gates were set based on the respective isotype controls (Cat.400612, APC Rat IgG2b, κ, BioLegend; Cat.400655, Brilliant Violet 421 Rat IgG2b, κ, BioLegend; Cat.400363, PE Rat IgG2b, κ, BioLegend; Cat.550085, PE Hamster IgG2, κ, BD). For live/dead staining the samples were co-stained with DAPI (Cat.422801, BioLegend) or Zombie Green fixable viability kit (Cat.423111, Biolegend). Cell populations were gated on living, single cells using FlowJo (Tree Star Inc.) software.

### 4.8 Immunohistochemistry

Tissues (ear skin, back skin, colon) were fixed overnight in 4% formaldehyde solution and washed in PBS. After fixation and paraffin-embedding, 5 μm (skin) or 7 μm (colon) sections were prepared. H&E staining and detection of phospho-RB (Cat.8516, Cell Signaling) was done as previously described (51).

### 4.9 Statistical analysis

Obtained results are either represented as the mean ± standard deviation (SD; *in vitro* stimulation) or mean ± SEM (standard error of the mean; n= animal numbers). Significance is depicted as asterisks (*P < 0.05, **P < 0.01, ***P < 0.001 and ****P< 0.0001) and was calculated based on 2-tailed Student’s t-test. All data plots were generated using GraphPad Prism software.

## Data availability statement

Publicly available datasets were analyzed in this study. This data can be found here: GDS4602 and GDS4600 (27).

## Ethics statement

The animal study was approved by Landesuntersuchungsamt Rheinland-Pfalz, Germany. The study was conducted in accordance with the local legislation and institutional requirements.

## Author contributions

BF: Formal analysis, Investigation, Project administration, Validation, Visualization, Writing – review & editing. TK: Formal analysis, Investigation, Writing – review & editing. AK: Formal analysis, Investigation, Writing – review & editing. JR: Formal analysis, Investigation, Visualization, Writing – review & editing. AW: Funding acquisition, Project administration, Resources, Supervision, Writing – review & editing. MW: Investigation, Writing – review & editing. SK: Funding acquisition, Project administration, Resources, Supervision, Writing – review & editing. SMK: Writing – review & editing. DK: Conceptualization, Formal analysis, Funding acquisition, Investigation, Project administration, Resources, Supervision, Validation, Visualization, Writing – original draft, Writing – review & editing.
