# Supplementary material for: IL-17A-driven psoriasis is critically dependent on IL-36 signaling
Source: Front Immunol. 2023 Dec 11;14:1256133. doi: 10.3389/fimmu.2023.1256133 (PMC10754973; doi:10.3389/fimmu.2023.1256133)
Supplement: Supplementary file 1 [file DataSheet_1.pdf]

Supplementary Figure 1

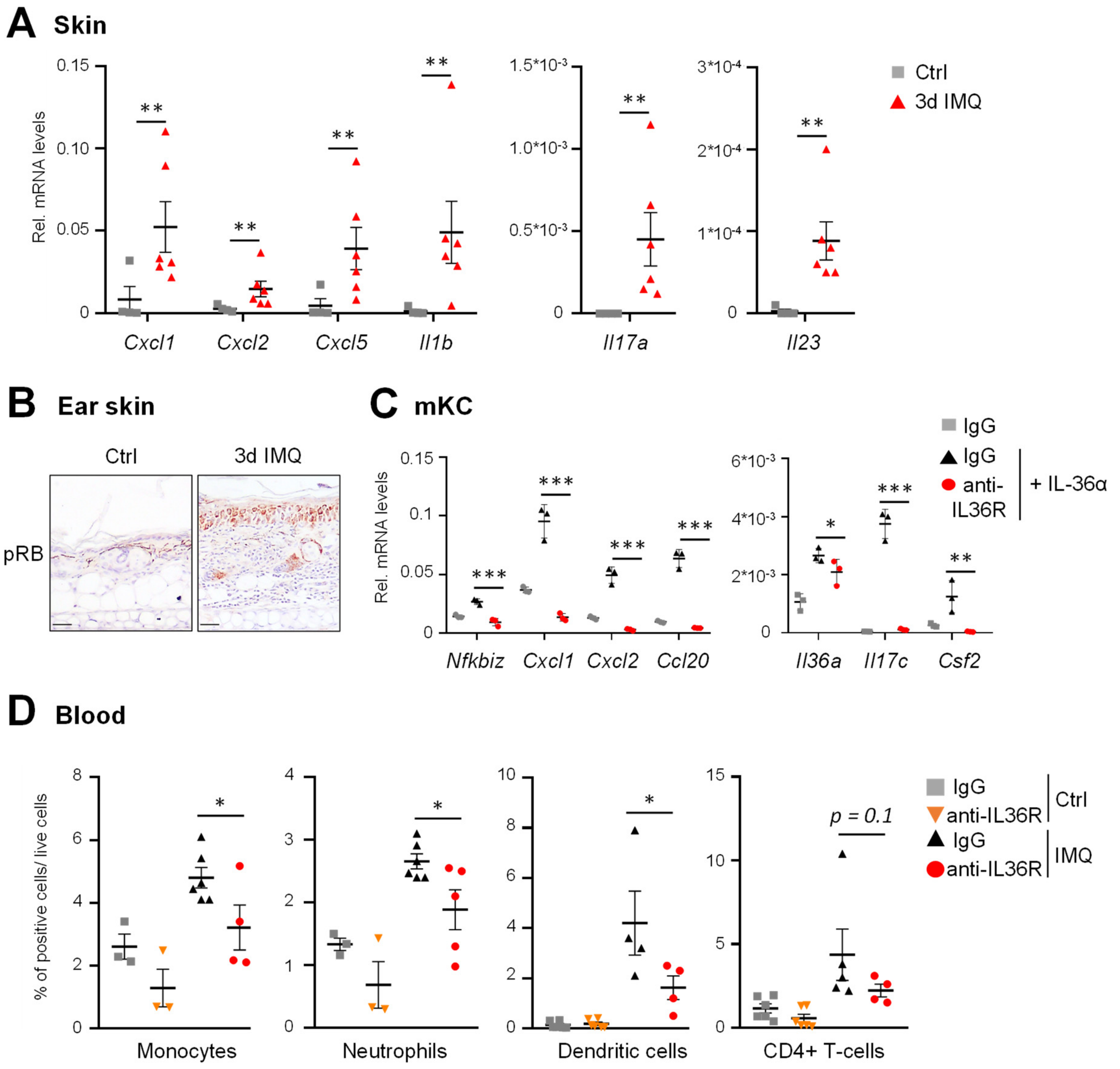

Supplementary Figure 1. Additional data of IMQ- and IgG- or anti-IL36R-treated animals. A. Relative mRNA expression levels of chemokines and cytokines in skin samples after 3 days of IMQ treatment, normalized to *β-Actin* ± SEM. **B.** pRB staining of ear skin after 3 days of IMQ treatment. Scale: 30 µm. **C.** Gene expression analysis of murine keratinocytes from Ctrl and IL-36α-treated cells in the presence of IgG or anti-IL36R (50 µg/mL), normalized to *β-Actin* ± SD. **D.** Flow cytometry analysis of immune cell populations in the blood of control or IMQ-treated animals in the presence of IgG or anti-IL36R (n = 3 - 6 animals per group). Shown is the relative percentage of positive cells, after pre-gating on viable cells. P-values were calculated using a 2-tailed Student's t-test (*P < 0.05, **P < 0.01, and ***P < 0.001).

Supplementary Figure 2

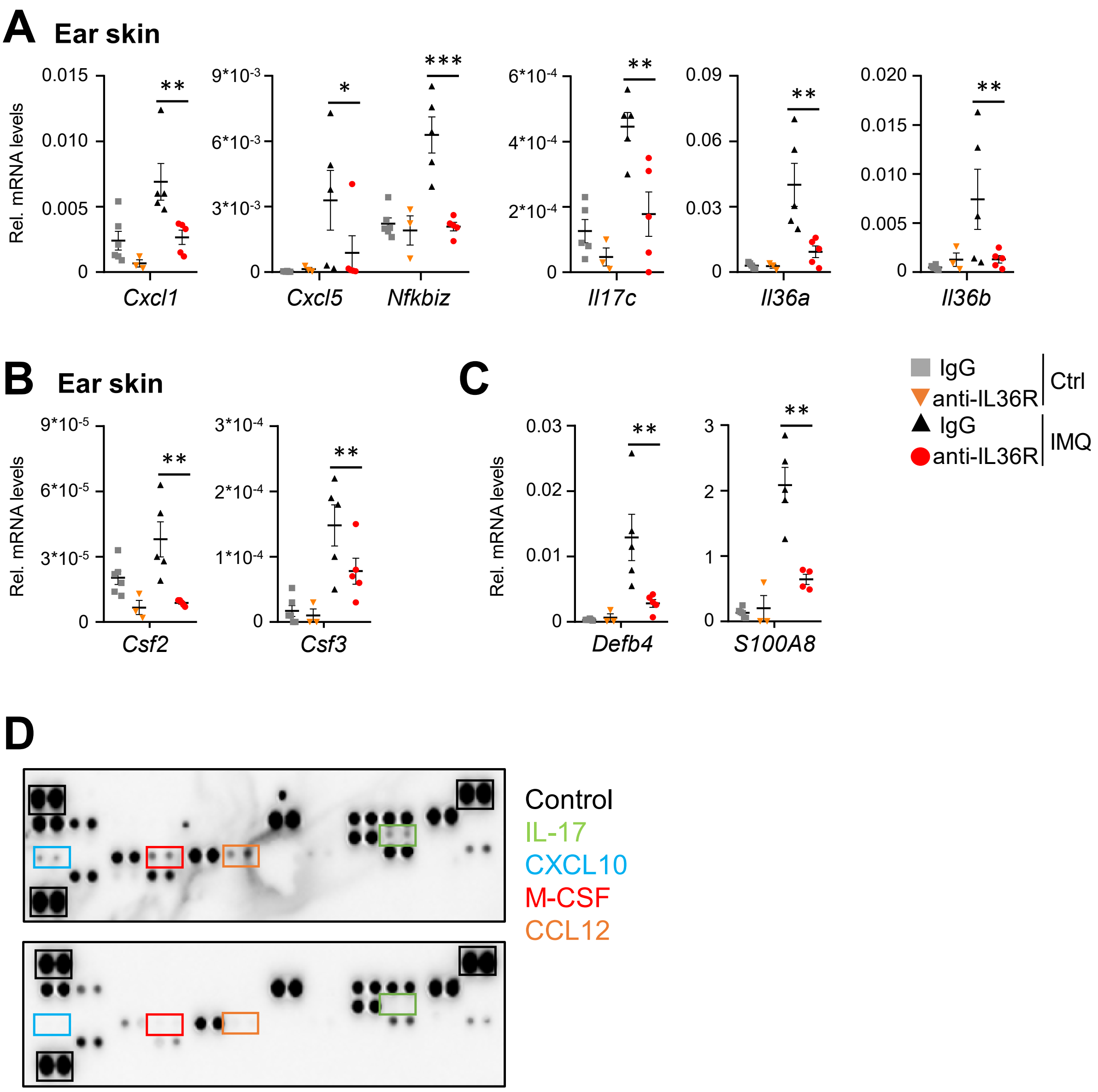

Supplementary Figure 2. Extended analysis of pro-inflammatory gene expression in IMQ and anti-IL36R-treated mice. A – C. Gene expression analysis of the skin from Ctrl or IMQ-treated animals, that received IgG or anti-IL36R treatment. Shown is the relative gene expression, normalized to *β-Actin*. n = 6 for Ctrl + IgG, n = 3 for Ctrl + anti-IL36R, n = 5 for IMQ + IgG or IMQ + anti-IL36R. **A.** Cytokines and psoriasis-associated co-factors. **B.** Growth factors. **C.** Anti-microbial peptides. Significance was calculated using a 2-tailed Student's t-test (*P < 0.05, **P < 0.01, and ***P < 0.001) showing the mean and standard error (SEM). **D.** Extended analysis of the cytokine array shown in Figure 3C. Shown is the same array with a longer exposition rate to visualize low-expressed proteins.

Supplementary Figure 3

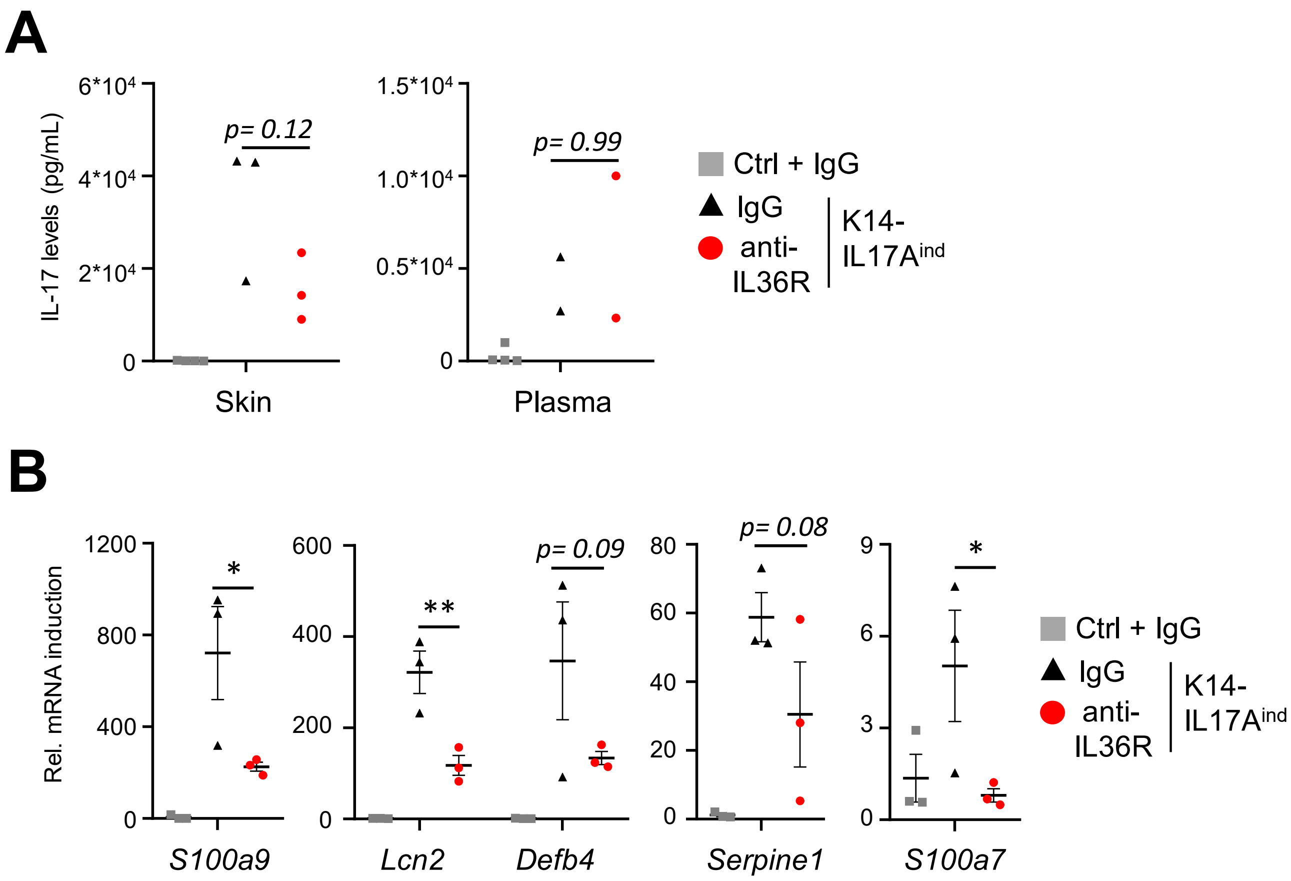

Supplementary Figure 3. Additional data on the effects of anti-IL36R treatment in K14-IL17A^{ind} mice. A. Quantification of IL-17A levels in skin or plasma of control and K14-IL17A^{ind} mice after receiving IgG or anti-IL36R treatment (n = 2-4 mice per group). **B.** Relative mRNA induction levels of antimicrobial peptides in skin lesions of IgG- and anti-IL36R-treated K14-IL17A^{ind} mice, normalized to *β-Actin* ± SD. P-values were calculated using a 2-tailed Student's t-test (*P < 0.05, **P < 0.01).

Supplementary Figure 4

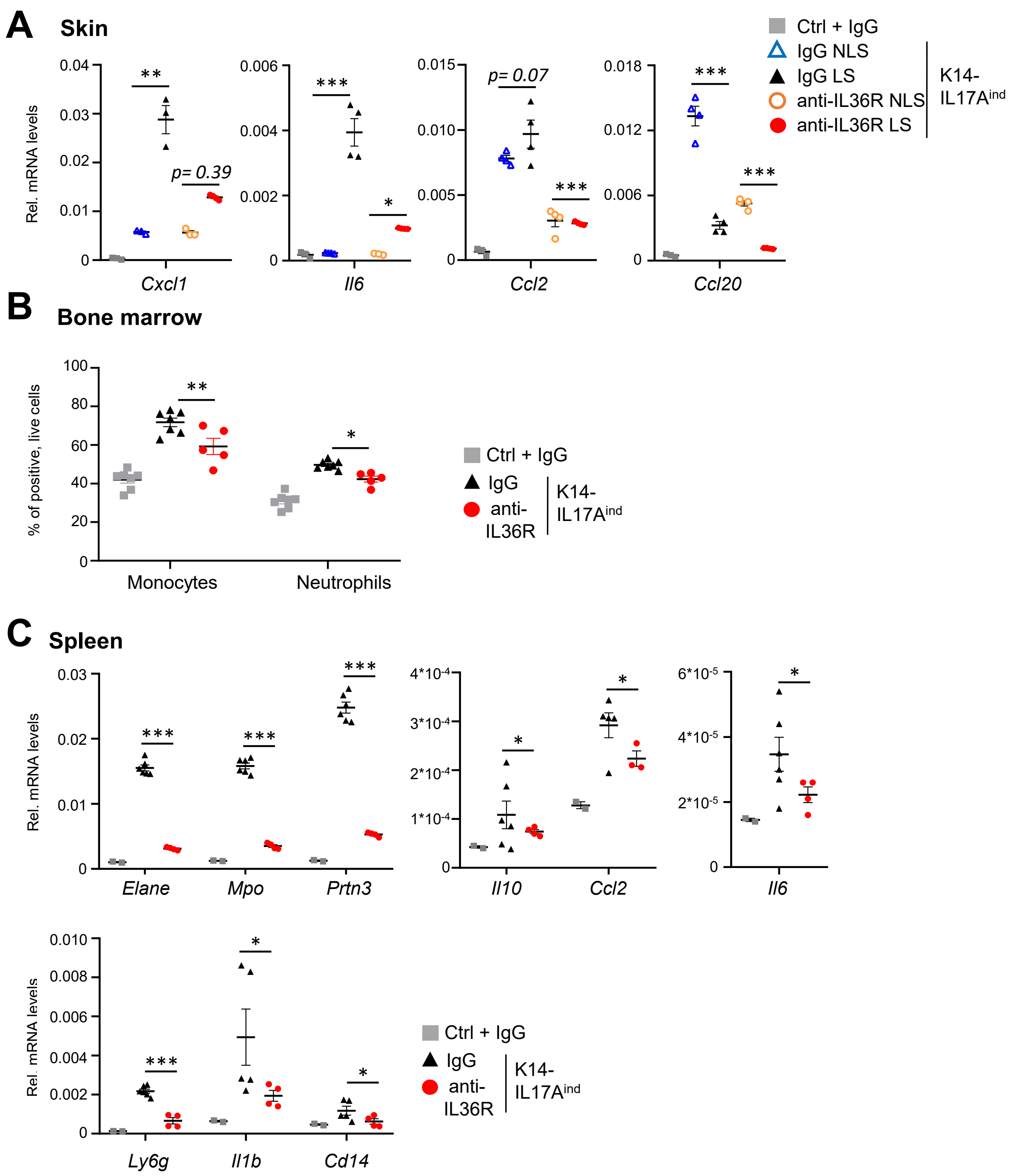

Supplementary Figure 4. Systemic effects of an acute anti-IL36R treatment in psoriatic K14-IL17A^{ind} mice. **A.** Relative mRNA expression levels of chemokines and cytokines in samples of lesional (LS) and non-lesional (NLS) skin from IgG-treated control and IgG- or anti-IL36R treated K14-IL17A^{ind} mice, normalized to *β-Actin*. n = 4 ± SEM. **B.** Flow cytometry analysis of monocytes and neutrophils in the bone marrow of control or K14-IL17A^{ind} mice, upon treatment with IgG or anti-IL36R antibodies (n = 5-7 animals per group). Shown is the relative percentage of positive cells, after pre-gating on viable cells. **C.** Relative mRNA expression levels of cytokines and neutrophil-specific markers in spleen samples obtained from IgG-treated control and IgG- or anti-IL36R treated K14-IL17A^{ind} mice, normalized to *β-Actin.* n = 3-5 ± SEM. All P-values were calculated using a 2-tailed Student's t-test (*P < 0.05, **P < 0.01, and ***P < 0.001).

Supplementary Table 1: Primer sequences for qPCR

Primer	Sequence 5' – 3'	Gene
mgActin_F	AGGAGTACGATGAGTCCGGC	*Actin*
mgActin_R	GGTGTAAAACGCAGCTCAGTA	
mgCcl2_F	CTGGAGCATCCACGTGTTGG	*Ccl2*
mgCcl2_R	CCCATTCCTTCTTGGGGTCAG	
mgCcl20_F	ACTACGACTGTTGCCTCTCG	*Ccl20*
mgCcl20_R	CTTGACTCTTAGGCTGAGGAGG	
mgCcl3_F	TTCTGCTGACAAGCTCACCC	*Ccl3*
mgCcl3_R	TCAACGATGAATTGGCGTGG	
mgCd14_F	ACTGAAGCCTTTCTCGGAGC	*Cd14*
mgCd14_R	AGCAACAGCAACAAGCCAAG	
mgCsf2_F	TCACGTTGAATGAAGAGGTAGAAG	*Csf2*
mgCsf2_R	ACTTGTGTTTCACAGTCCGTTTC	
mgCsf3_F	ATCCATGGCTCAACTTTCTGC	*Csf3*
mgCsf3_R	GCTGCAGGGCCATTAGCTTC	
mgCxcl1_F	ACGTGTTGACGCTTCCCTTG	*Cxcl1*
mgCxcl1_R	TCCTTTGAACGTCTCTGTCCC	
mgCxcl2_F	CGCCCAGACAGAAGTCATAGC	*Cxcl2*
mgCxcl2_R	CTTTGGTTCTTCCGTTGAGGG	
mgCxcl5_F	CCCTACGGTGGAAGTCATAGC	*Cxcl5*
mgCxcl5_R	GAACACTGGCCGTTCTTTCC	
mgDefb4_F	GGTGCTGCTGTCTCCACTTG	*Defb4*
mgDefb4_R	TATTCATCTTGCTGGTTCTTCGTC	
mgElane_F	ACCCAGTGTGCTACAAGAGC	*Elane*
mgElane_R	GTGCATACGTTCACACGACG	
mgIfng_F	GACAATCAGGCCATCAGCAAC	*Ifng*
mgIfng_R	CATTGAATGCTTGGCGCTGG	
mgIl17a_F	GCCCTCAGACTACCTCAACC	*Il17a*
mgIl17a_R	TTCCCTCCGCATTGACACAG	
mgIl17c_F	GGAGACAGCATGAAGGACCTC	*Il17c*
mgIl17c_R	GCTTCTGTGGGTAGCGGTTC	
mgIl17f_F	TGCTACTGTTGATGTTGGGAC	*Il17f*
mgIl17f_R	AATGCCCTGGTTTTGGTTGAA	
mgIl1b_F	AGCTGAAAGCTCTCCACCTC	*Il1b*
mgIl1b_R	GCTTGGGATCCACACTCTCC	
mgIl22_F	CCTACATGCAGGAGGTGGTG	*Il22*
mgIl22_R	CCCAATCGCCTTGATCTCTCC	
mgIl23a_F	CAGCTCTCTCGGAATCTCTGC	*Il23a*
mgIl23a_R	TGTCCTTGAGTCCTTGTGGG	
mgIl33_F	CTCACTGCAGGAAAGTACAGC	*IL33*
mgIl33_R	TGGTCTTCTGTTGGGATCTTC	
mgIl36a_F	GCCTGTTCTGCACAAAGGATG	*Il36a/Il1f6*
mgIl36a_R	ACAGCGATGAACCAACCAGG	
mgIl36b_F	AGGGCAAACCAACTTTGCAG	*Il36b*

mgIl36b_R	GAAGTGGAGCCCTCTATGCC	
mgIl36g_F	GTCAGCGTGACTATCCTCCC	*Il36g/Il1f8*
mgIl36g_R	TGGCTTCATTGGCTCAGGG	
mgIl1rl2_F	TTACGCGACACAGCTTGTTTG	*Il1rl2/IL36R*
mgIl1rl2_R	TCACACGTATCTGCTGCCAC	
mgIl6_F	GTCCGGAGAGGAGACTTCAC	*Il6*
mgIl6_R	GCAAGTCGATCATCGTTGTTC	
mgLcn2_F	AATGTCACCTCCATCCTGGTC	*Lcn2*
mgLcn2_R	ACTGGTTGTAGTCCGTGGTG	
mgLy6g_F	ACCTGAGACTTCCTGCAACAC	*Ly6g*
mgLy6g_R	CACGTTGACAGCATTACCAGTG	
mgMpo_F	GGAGGCCCGGAAGATTGTAG	*Mpo*
mgMpo_R	CGTTGGTGAAGACATTGGCG	
mgNfkbiz_F	AACTCGCCAAGAGACCAGTG	*Nfkbiz*
mgNfkbiz_R	AGAGCCACTGACTTGGAACG	
mgNos2_F	ACAGGGCTACAACATCCTGG	*Nos2*
mgNos2_R	GACTCTGAGGCTGTGTGGTG	
mgPrtn3_F	CAGCTAAACCGGACAGCCTC	*Prtn3*
mgPrtn3_R	GTTCCCGGCATAGGAAGGTG	
mgRpl37a_F	CCAAGGCCTACCACCTTCAG	*Rpl37a*
mgRpl37a_R	CTTGGGTTTCGGCGTTGTTC	
mgS100A7_F	TCTGCTCTTGGATAGTGTGCC	*S100a7*
mgS100A7_R	TGATGTAGTATGGCTGCCTGC	
mgS100A9_F	AATGGTGGAAGCACAGTTGG	*S100a9*
mgS100A9_R	CTGGTTTGTGTCCAGGTCCTC	
mgSerpine1_F	CAAATGGTGGCCCAATAGCG	*Serpine1*
mgSerpine1_R	AGCAAGCTGTGTCAAGGGAG	
mgTnfa_F	AAGTTCCCAAATGGCCTCCC	*Tnfa*
mgTnfa_R	TTGCTACGACGTGGGCTAC	
